# Real-world clinical outcomes with avelumab in patients with Merkel cell carcinoma treated in the USA: a multicenter chart review study

**DOI:** 10.1136/jitc-2022-004904

**Published:** 2022-08-18

**Authors:** Shailender Bhatia, Paul Nghiem, S Phani Veeranki, Alejandro Vanegas, Kristina Lachance, Lisa Tachiki, Kevin Chiu, Emily Boller, Murtuza Bharmal

**Affiliations:** 1 Division of Medical Oncology, University of Washington, Seattle, Washington, USA; 2 Fred Hutchinson Cancer Research Center, Seattle, Washington, USA; 3 UW Medical Center at Lake Union, Seattle, Washington, USA; 4 PRECISIONheor, Los Angeles, California, USA; 5 RTI Health Solutions, Parsippany, New Jersey, USA; 6 EMD Serono Research & Development Institute, Inc, Billerica, Massachusetts, USA, an affiliate of Merck KGaA

**Keywords:** immunotherapy, skin neoplasms

## Abstract

**Background:**

Merkel cell carcinoma (MCC) is a rare, aggressive, cutaneous neuroendocrine neoplasm with annual incidence rates of 0.13–1.6 cases/100,000/year worldwide as of 2018. Chemotherapy for metastatic MCC (mMCC) has high objective response rates (ORRs), but responses are not durable and overall survival (OS) is poor. Avelumab (anti-programmed death-ligand 1) has demonstrated meaningful survival benefit and durable responses in clinical trials for mMCC. This study investigated real-world clinical outcomes in avelumab-treated patients with advanced (stage IIIB/IV) MCC in US academic medical centers.

**Methods:**

We conducted a retrospective chart review of patients with advanced MCC who initiated avelumab between March 1, 2017, and July 31, 2019, at six US academic centers. Data were requested for eligible patients from index date through December 31, 2020. Descriptive analyses were conducted to assess demographic and clinical characteristics, real-world ORR (rwORR), real-world duration of response, real-world progression-free survival (rwPFS), and OS.

**Results:**

Ninety patients with advanced MCC (82%, stage IV; 18%, stage IIIB) received avelumab. Median follow-up was 20.8 months (95% CI: 19.1 to 24.2). Median age was 68 years (range, 48–83), and the majority of patients were men (58%) and white (93%). The primary tumor was most commonly located on the lower limb (38%), with metastases mostly located in lymph nodes (68%), lung (52%), and viscera (52%). Approximately 42% and 26% of patients had an Eastern Cooperative Oncology Group performance status of 2 and 3, respectively. Seventy-three patients (81%) received avelumab as first-line treatment of advanced MCC, while 17 (19%) received avelumab as second-line or later treatment. The median duration of avelumab treatment was 13.5 months (95% CI: 6.4 to 30.6), with 42% of patients still receiving avelumab by the end of follow-up. Patients with avelumab treatment had an rwORR of 73% (95% CI: 64 to 83), median rwPFS of 24.4 months (95% CI: 8.31 to not estimable (NE)), and median OS of 30.7 months (95% CI: 11.2 to NE).

**Conclusions:**

This real-world study of patients with advanced MCC demonstrated that avelumab treatment resulted in a high response rate with durable responses and prolonged survival. The study findings validate the results demonstrated in prospective clinical trials and other observational studies.

**WHAT IS ALREADY KNOWN ON THIS TOPIC**

- Advanced Merkel cell carcinoma (MCC) is an aggressive rare form of skin cancer with significant morbidity and mortality.
- Avelumab (anti-programmed death-ligand 1) has demonstrated meaningful survival benefit and durable responses in clinical trials for patients with metastatic MCC.

**WHAT THIS STUDY ADDS**

- Typically, real-world clinical practice patient populations tend to be broader than those defined by the relatively restricted clinical trial eligibility criteria.
- This real-world study of patients with advanced MCC demonstrated that avelumab treatment resulted in a high response rate with durable responses and prolonged survival, validating the results in prospective clinical trials and other observational studies.

**HOW THIS STUDY MIGHT AFFECT RESEARCH, PRACTICE OR POLICY**

- This study generates complementary data on the effectiveness of avelumab in real-world settings, with new evidence in patients with stage IIIB MCC and among those with a more severe Eastern Cooperative Oncology Group performance status, which will further inform clinicians in their treatment decision-making.

## Background

Merkel cell carcinoma (MCC) is a rare skin cancer that usually presents as painless red or flesh-colored nodule(s) on the skin, most commonly on the face, head, or neck.^1^ Risk factors for MCC include sun exposure, immunodeficiency (specifically T-cell dysfunction), and the presence of Merkel cell polyomavirus.^2^ MCC is becoming increasingly common, with an incidence that tripled over the past two decades in the USA and an estimated 2835 new cases per year in 2020 and a forecasted incidence of 3284 cases per year by 2025.^2 3^ The significant increase can be attributed in part to better awareness and improved diagnostic techniques but largely to the increase in the size of the older adult population with extensive sun exposure during their lifetimes.^4^ MCC has a mortality rate of 30%, which is more lethal than that of malignant melanoma.^5^ Patients with local disease had a 64% relative survival at 5 years, compared with the 39% among patients with regional nodal disease and 18% among patients with metastatic disease.^6^ The prognosis of MCC is often poor because of its rapid growth, potential for metastasis, and high rates of recurrence.^7^

The choice of treatment for MCC often depends on the stage of the disease. Treatments generally include surgery, radiotherapy, or both for patients with localized and regional disease and immunotherapy or chemotherapy for patients with advanced disease. The National Comprehensive Cancer Network guidelines recommend treatment with pembrolizumab for locally advanced disease and treatment with avelumab, pembrolizumab, or nivolumab for disseminated MCC.^8^ Avelumab, a human IgG1 anti-programmed death-ligand 1 (PD-L1) monoclonal antibody, was approved under accelerated approval by the US Food and Drug Administration in March 2017 as the first treatment for patients with metastatic MCC (mMCC).^9^ This approval was based on data from the open-label, single-arm, multicenter clinical trial JAVELIN Merkel 200, which demonstrated a clinically meaningful and durable objective response rate of 33% in previously treated patients with mMCC.^9–11^ Median overall survival (OS) was 12.6 months, the 3-year survival rate was 32%, the 4-year survival rate was 30%, and the 5-year survival rate was 26% in the JAVELIN Merkel 200 clinical trial.^10 12^

Although avelumab has demonstrated significant improvement in efficacy and safety among patients with MCC in clinical trials and expanded access programs,^9–13^ the treatment patterns and associated outcomes with avelumab in patients with MCC in real-world settings are less understood. While endpoints from clinical trials are considered the gold standard in clinical evidence, the relatively restricted eligibility criteria, controlled setting, and sample size of clinical trials may limit the generalizability of their findings to the broader real-world population.^14^ For example, real-world data collection does not often follow the same standard or process as clinical trials, as evidence collected in the real world is often abstracted from unstructured documents or inferred through physician responses or laboratory results. Real-world studies are fundamental to understanding the effectiveness of avelumab in clinical practice for the treatment of patients with MCC. Real-world studies can provide evidence of which patient types are granted access to and receive treatment, how they tolerate the drug in practice, and how effective the drug is in real-world settings.

The goal of this study was to assess the characteristics, current treatment patterns, and real-world clinical outcomes of patients with stage IIIB/IV MCC who initiated avelumab in participating academic medical centers and satellite clinics of academic institutions in the USA. This study’s primary endpoints are real-world objective response rate (rwORR), real-world duration of response (rwDOR), real-world progression-free survival (rwPFS), and OS.

## Methods

### Study design, data source, and patient population

**Figure 1:**
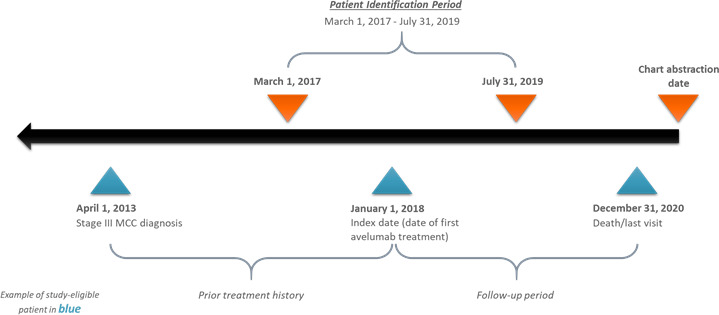
Study design schematic. MCC, Merkel cell carcinoma.

This was a retrospective, observational, medical chart review study of patients who initiated treatment with avelumab (index date) for stage IIIB/IV MCC from March 1, 2017, to July 31, 2019 (patient identification period). Data were requested for eligible patients from the initial diagnosis of advanced MCC until December 31, 2020 (figure 1). Participating academic medical centers and satellite clinics were randomly selected from an American Medical Association database and from an internal Medical Data Analytics (now RTI Health Solutions) research database. Oncologists, dermatologists, and other similar and related specialists at the selected centers were asked to fill out a physician screening questionnaire to assess eligibility. The physicians were screened to determine eligible patient caseloads and to ensure agreement with study protocols. To minimize selection bias and avoid convenience sampling, the physicians were provided with a list of randomly generated month–year time points between March 1, 2017, and July 31, 2019, to identify study-eligible patients in the centers. Physicians identified patients with MCC who initiated avelumab at the provided month–year. If they did not find any eligible patients in the given month, additional month–year time points were provided until the providers identified their allocated eligible patients for the study. Using the patients’ electronic medical records, physicians and their respective site-specific staff completed case report forms to provide demographic and clinical information on avelumab-treated patients with stage IIIB/IV MCC. Specifically, the case report forms included information on comorbidities, initial MCC diagnosis and staging, adjuvant and neoadjuvant therapies received, advanced MCC diagnosis and staging, each therapy used to treat advanced MCC, and disease status during the most recent follow-up. Ongoing follow-ups were made with recruited physicians to address any questions that arose throughout the study. Any discrepant data on any patient case report form resulted in recontacting the physician for clarification and resolution.

The study included adult patients (≥18 years of age at diagnosis) with stage IIIB/IV MCC (in alignment with the American Joint Committee on Cancer's staging system^15^) at the initiation of avelumab treatment between March 1, 2017, and July 31, 2019 (index date). Patients with other primary cancers or treatments for other primary cancers in the 3 years prior to and including the index date were excluded (except for basal or squamous cell carcinoma of the skin, bladder carcinoma in situ, cervical carcinoma in situ, chronic lymphocytic leukemia, multiple myeloma, or hypogammaglobulinemia).

### Variables and analyses

Our primary endpoints of interest were rwORR, rwDOR, rwPFS, and OS. Prospective clinical trial endpoints often use Response Evaluation Criteria in Solid Tumors (RECIST) as a standard to determine progression and tumor response, which can be independently confirmed by central reviewers.^16^ Applying strict RECIST is not always feasible with retrospective real-world data.^14 16^ Therefore, unlike OS, which has a standard mortality event, the endpoints of ORR, PFS, and DOR may fall under a different standard in a real-world scenario versus a clinical trial scenario. This study distinguishes real-world endpoints by affixing ‘rw’ to ORR, PFS, and DOR, as real-world endpoints can have different estimates than their clinical trial counterparts.^17^

The rwORR was calculated as the number of patients who achieved either a complete response (CR) or partial response (PR) divided by the total number of patients in the study. For assessing tumor response, physicians abstracting the data were asked to indicate each patient’s best response to therapy from their medical chart narrative into CR, PR, stable disease (SD), or progressive disease (PD). As is typical with real-world evidence studies, tumor response assessment occurred in an unstructured way and was physician-reported without independent confirmation by central reviewers. The rwDOR outcome was calculated for each patient as the duration of time from first documented CR or PR to the earliest date of first progression or recurrent disease or date of death. The OS variable was defined as the interval (in months) between the treatment start date and the date of death, as documented in the patient’s case report form. The rwPFS was measured from the index date of treatment to the date of progression or date of death due to any cause. Patients who were still alive and did not have progressive disease at the last office visit without clinical or radiographic evidence of progression were censored. Time until treatment discontinuation (TTD) measured the length of time between avelumab initiation and discontinuation. To estimate the length of time for both TTD and follow-up time, a reverse Kaplan-Meier estimator was used. This method defined discontinuation of avelumab as an event, and the time to discontinuation was measured. All individuals who died while being treated with avelumab were censored.^18^

Descriptive analyses were conducted to assess demographics, clinical characteristics, treatment patterns, and outcomes during follow-up. The descriptive analysis was further stratified by the number of systemic therapies and lines of therapy. Kaplan-Meier curves were constructed to estimate censor-adjusted study outcomes, including rwDOR, OS, and rwPFS with medians and 95% CIs. Additional Kaplan-Meier curves were constructed and stratified by line of therapy and stage of MCC at the time of avelumab initiation/index date.

## Results

### Site and patient characteristics

#### Site characteristics

A total of 925 physicians were contacted to assess participation interest and eligibility. Of those contacted, 79 physicians responded and 38 were interested. Of those interested, 10 physicians across 10 sites were eligible to participate in the current study; 6 physicians ultimately agreed to participate. Two sites were located in the Northeast, 2 in the Midwest, 1 in the West, and 1 in the South. Sixty per cent of the patient charts originated from 2 sites, and the remaining 4 sites provided 40% of the charts.

#### Patient characteristics

The study population comprised 90 adult patients diagnosed with stage IIIB/IV MCC who initiated treatment with avelumab between March 1, 2017, and July 31, 2019. The median age of patients was 68 years (range, 48–83 years) (table 1). The majority were men (57.8%), white (93.3%), non-Hispanic (98.9%), and covered by commercial/private insurance (47.8%). The stage of MCC at initiation of avelumab was IIIB in 16 patients (18%) and IV in 74 patients (82%), respectively. The lower limb/trunk was the most common primary location of tumors (38%). Forty-seven patients (52%) had a visceral metastasis, with the lymph nodes (67%) and lungs (55%) as the primary sites for tumor metastasis. Forty-three patients (48%) were tested for the expression of Merkel cell polyomavirus; of those, 34 (79%) had tumors that were positive. Thirty-five patients (39%) were assessed for PD-L1 expression; of these patients, 32 (91%) had positive PD-L1 expression.

**Table 1:** Patient demographics and clinical characteristics

Patient characteristics* N=90	
Age, mean (SD) [median], years	67.1 (8.0) [68]
Male, n (%)	52 (57.8)
Female, n (%)	38 (42.2)
Race and ethnicity, n (%)	
White	84 (93.3)
African American	4 (4.4)
Asian	1 (1.1)
American Indian/Alaskan Native	4 (1.1)
Non-Hispanic, n (%)	89 (98.9)
Height, mean (SD), cm	168.7 (10.9)
Weight, mean (SD), kg	76.6 (11.6)
Medical/drug insurance, n (%)	
Commercial/private	43 (47.8)
Medicaid	9 (10.0)
Medicare	38 (42.2)
Tumor characteristics*	**n (%)**
Stage of diagnosis at the time of avelumab initiation	
IIIB	16 (17.8)
IV	74 (82.2)
Location of primary tumor†	
Face	8 (8.9)
Lower limb/trunk	34 (37.8)
Scalp and neck	27 (30)
Upper limb	20 (22.2)
Unknown	5 (5.6)
Other	1 (1.1)
Sites of distant metastasis‡	
Lymph nodes	60 (66.7)
Distant skin	24 (26.7)
Lung	47 (52.2)
Bone	14 (15.6)
Liver	31 (34.4)
Brain	1 (1.1)
Other	2 (2.2)
Merkel cell polyomavirus	
Positive	34 (37.8)
Negative	9 (10.0)
Unknown	47 (52.2)
PD-L1 status	
Positive	32 (35.6)
Negative	3 (3.3)
Unknown	55 (61.1)
ECOG PS	
0	0
1	28 (31.1)
2	38 (42.2)
3	23 (25.6)
4	1 (1.1)
5	0
**Comorbid conditions‡**	**n (%)**
Any comorbidity	41 (45.6)
Individual comorbidities	
AIDS (not only HIV positive)	1 (2.4)
Any prior solid tumor (within 5 years of diagnosis)	1 (2.4)
Autoimmune condition, other	2 (4.8)
Cerebrovascular disease	6 (14.3)
Chronic obstructive pulmonary disease	11 (26.2)
Congestive heart failure	7 (16.7)
Connective tissue disease	3 (7.1)
Coronary artery disease	16 (38.1)
Dementia	1 (2.4)
Diabetes with organ damage	5 (11.9)
Diabetes (no complications)	10 (23.8)
Hemiplegia or paraplegia	0
Hepatitis	0
Leukemia	1 (2.4)
Lymphoma	0
Mild liver disease	3 (7.1)
Moderate-to-severe liver disease	1 (2.4)
Moderate-to-severe renal disease	2 (4.8)
Peptic ulcer disease	2 (4.8)
Peripheral vascular disease	5 (11.9)
Other	1 (2.4)

*On index date.

†Not mutually exclusive.

‡Pre-index period.

ECOG PS, Eastern Cooperative Oncology Group performance status; PD-L1, programmed death-ligand 1.

This study used Eastern Cooperative Oncology Group performance status (ECOG PS)^19^ as one measure of the patient’s level of functioning. The majority (69%) of the patients in this real-world study had an ECOG PS score of higher than 1: 26% had an ECOG PS of 3, 42% had an ECOG PS of 2, and 1% had an ECOG PS of 4. Forty-one patients (46%) were reported to have ≥1 comorbidity during the pre-index period, and three of those patients were immunocompromised.

### Avelumab treatment characteristics

Of the 90 total patients who received avelumab, 86 received avelumab only, while 4 received avelumab plus carboplatin (table 2). Of patients receiving carboplatin, all 4 had an ECOG PS of 1, and 3 had stage IV MCC at the start of avelumab initiation. None had visceral or bone metastases.

**Table 2:** Avelumab treatment characteristics

Treatment on the index date	Patients, n	Follow-up period, median (95% CI), months	Treatment discontinuation (avelumab), n (%)	TTD (avelumab), median (95% CI), months
Avelumab only or avelumab+systemic therapy	90	20.8 (19.1 to 24.2)	52 (57.8)	13.5 (6.4 to 30.6)
Avelumab only	86	20.8 (19.1 to 24.2)	52 (60.5)	12.0 (9.3 to 30.5)
Avelumab+systemic therapies (carboplatin and etoposide)	4	17.5 (17.4 to NE)	0	NE (NE to NE)
Patients received avelumab as 1L treatment	73	21.3 (19.6 to 25.3)	38 (52.1)	24.5 (7.4 to 37.7)
Patients received avelumab as 2L+ treatment	17	18.17 (12.0 to 20.8)	14 (92.4)	3.4 (1.8 to 13.5)

1L, first line; 2L+, second or later line; NE, not estimable; TTD, time until treatment discontinuation.

Seventy-three patients (81%) received avelumab as first-line (1L) treatment for advanced MCC, while 17 (19%) received avelumab as second-line or later (2L+) treatment. Of these 17 patients, 2 patients received only one therapy (carboplatin or cisplatin), and 15 patients received two therapies—etoposide plus carboplatin (12 patients), etoposide plus cisplatin (2 patients), or paclitaxel plus carboplatin (1 patient).

Among all avelumab-treated patients with MCC, the median follow-up was 20.8 months (95% CI: 19.1 to 24.2). More than half of patients (58%) discontinued avelumab during the study period, and the median TTD was 13.5 months (table 2).

### Avelumab treatment outcomes

#### rwORR

Of the 90 patients enrolled, 35 (39%) had a CR, while 31 (34%) had a PR, resulting in an rwORR of 73% (95% CI: 64.0 to 82.6). The rwORR in patients receiving only avelumab was 72.1% (95% CI: 62.4 to 81.7). Of the patients who were treated with avelumab plus other systemic therapies, three had a CR and one had a PR, with rwORR of 100%. Of the patients who received avelumab as 1L treatment, 45% had a CR and 30% had a PR, resulting in an rwORR of 75% (95% CI: 65.2 to 85.4). In patients receiving avelumab as 2L+ treatment, the rwORR was 64.7% (95% CI: 39.4 to 90.0) (table 3).

**Table 3:** Response to avelumab treatment

Treatment on the index date	Avelumab only or avelumab +systemic therapy (N=90)	Avelumab only (n=86)	Avelumab+systemic therapies (n=4)	Avelumab as 1L treatment (n=73)	Avelumab as 2L+ treatment (n=17)
rwORR (95% CI), %	73.3 (64.0 to 82.6)	72.1 (62.4 to 81.7)	100 (N/A)	75.3 (65.2 to 85.4)	64.7 (39.4 to 90.0)
Response to treatment, n (%)					
CR	35 (38.9)	32 (37.2)	3 (75.0)	33 (45.2)	2 (11.7)
PR	31 (34.4)	30 (34.9)	1 (25.0)	22 (30.1)	9 (52.9)
SD	5 (5.6)	5 (5.8)	0	3 (4.1)	2 (11.7)
PD	19 (21.1)	19 (22.0)	0	15 (20.5)	4 (23.5)

CR, complete response; N/A, not applicable; PD, progressive disease; PR, partial response; rwORR, real-world objective response rate; SD, stable disease.

When stratified by stage of MCC, 16 patients had stage IIIB disease (data not shown in table). Of these, 14 (87.5%) had a CR and 2 (12.5%) had a PR, with a 100% rwORR. Of the 74 patients who were diagnosed with stage IV disease at the time of avelumab initiation, 28.5% achieved a CR and 39.2% achieved a PR, resulting in a rwORR of 67.6% (95% CI: 57.6 to 79.4).

#### rwDOR

**Figure 2:**
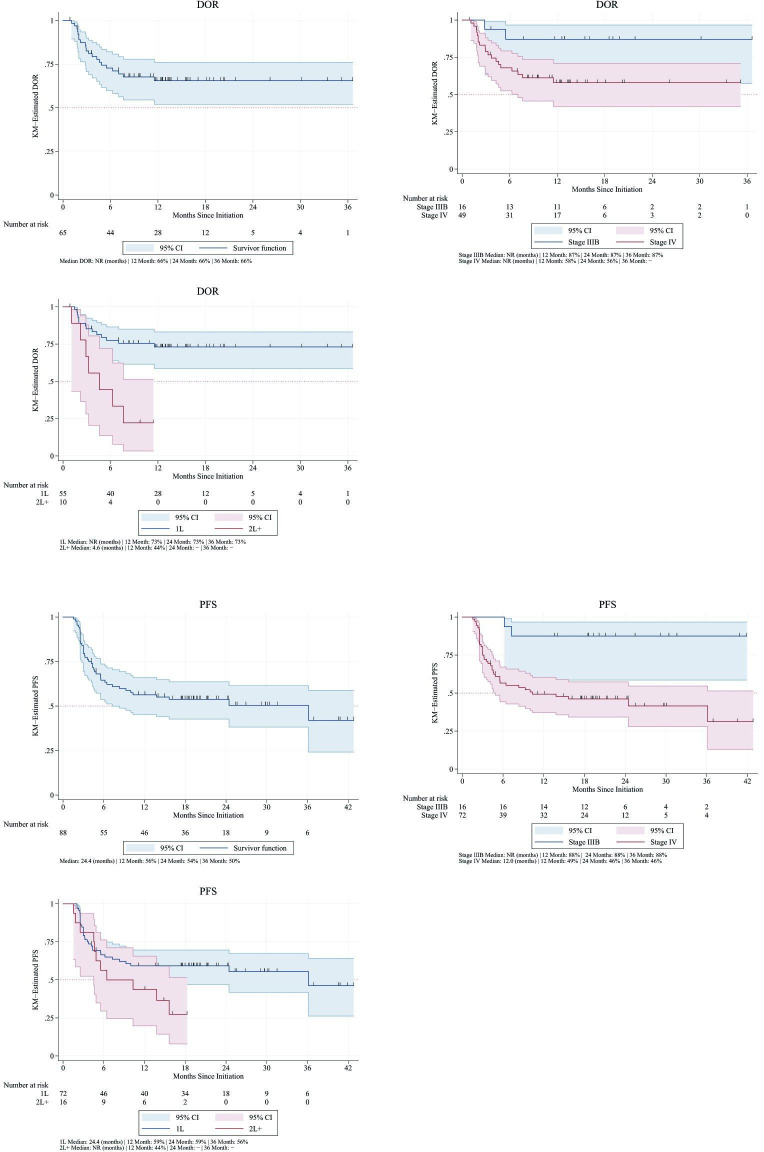
Real-world duration of response (DOR) and real-world progression-free survival (PFS) among all avelumab-treated patients, by stage of avelumab initiation and by line of avelumab treatment. KM, Kaplan-Meier; NR, not reached; 1L, first line; 2L+, second or later line.

The median rwDOR for all avelumab-treated patients and most of our subgroups of interest (avelumab only, avelumab in combination with other systemic therapy, avelumab by stage of MCC, and avelumab as 1L therapy) was not estimable (NE) due to patients being censored given event non-occurrence (ie, ongoing responses, so the ‘end’ of response had not been met). Median rwDOR was estimated to be 4.6 months (95% CI: 1.1 to NE) in patients receiving avelumab as a 2L+ treatment (table 4). The rwDOR rates at 24 months in patients with stage IIIB and stage IV MCC were 87% and 58%, respectively. In patients with 1L avelumab use, the rwDOR rate at 24 months was 73% (figure 2).

**Table 4:** Real-world clinical outcomes among avelumab-treated patients with advanced MCC

Treatment on index date	Avelumab or avelumab +systemic therapy (N=90)	Avelumab only (n=86)	Avelumab+systemic therapies (n=4)	Patients received avelumab as 1L treatment (n=73)	Patients received avelumab as 2L+ treatment (n=17)
rwDOR	NE (NE to NE)	NE (NE to NE)	NE (NE to NE)	NE (NE to NE)	4.6 (1.1 to NE)
rwPFS	24.4 (8.3 to NE)	24.4 (6.4 to NE)	NE (NE to NE)	36.1 (9.3 to NE)	6.4 (4.5 to NE)
OS	30.7 (11.2 to NE)	25.7(9.7 to NE)	NE (NE to NE)	41.7 (10.2 to NE)	15.9 (4.3 to NE)

Estimates represent median (95% CI) in months.

1L, first line; 2L+, second or later line; MCC, Merkel cell carcinoma; NE, not estimable; OS, overall survival; rwDOR, real-world duration of response; rwPFS, real-world progression-free survival.

#### rwPFS

The median rwPFS among all avelumab-treated patients was 24.4 months (8.3–NE). In patients treated with avelumab only, median rwPFS was 24.4 months (6.4–NE). Median rwPFS was NE for patients treated with avelumab and systemic therapies due to patients being censored given event non-occurrence. Median rwPFS was also NE for patients with stage IIIB MCC. However, patients with stage IV disease had a median rwPFS of 12.0 months (4.9–NE). Those receiving avelumab as a 1L therapy had a median rwPFS of 36.1 months (9.3–NE), whereas those who received avelumab in the 2L+ setting had a median rwPFS of 6.4 months (4.5–NE) (table 4).

At 24 months, the rwPFS rate was 54% in all patients who received avelumab. The rwPFS rates at 24 months in patients with stage IIIB and stage IV disease were 88% and 46%, respectively. In patients who received 1L avelumab, the rwPFS rate at 24 months was 59% (figure 2).

#### OS

**Figure 3:**
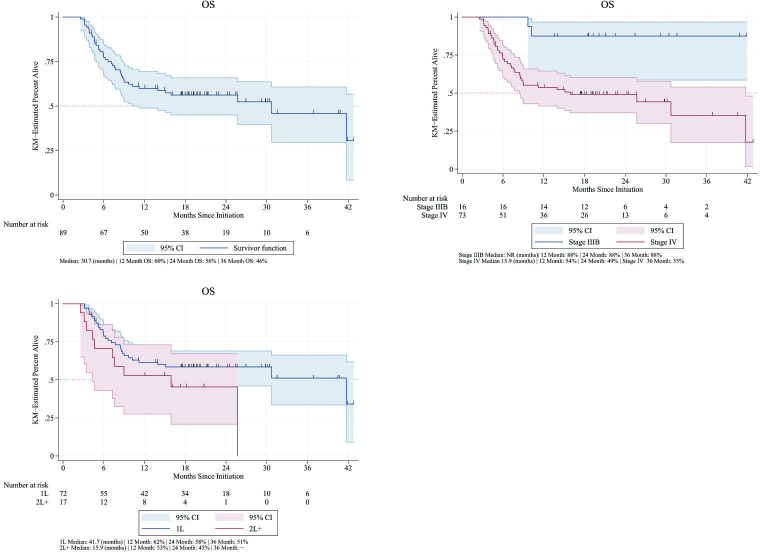
Overall survival (OS) among all avelumab-treated patients, by stage of avelumab initiation and by line of avelumab treatment. KM, Kaplan-Meier; 1L, first line; 2L+, second or later line.

The median OS among all avelumab-treated patients was 30.7 months (11.2–NE). In patients treated with avelumab only, the median OS was 25.7 months (9.7–NE). The median OS in the patients with stage IIIB disease and those who received avelumab in combination with other therapies was NE. The median OS in all avelumab-treated patients with stage IV MCC was 15.9 months (4.3–NE) (figure 3). Patients who received avelumab in the 1L tended to have a better prognosis, as evidenced by their higher median OS of 41.7 months (10.2–NE). Patients who were treated with 2L+ therapy had a median OS of 15.9 months (table 4).

In all avelumab-treated patients, the OS rate at 24 months was 56%. The OS rates at 24 months in patients with stage IIIB and stage IV MCC were 88% and 49%, respectively. In patients with 1L avelumab use, the OS rate at 24 months was 58%, while in those with 2L+ use, the OS rate at 24 months was 45% (figure 3). All deaths in the study were reported as MCC-related deaths.

### Treatment after avelumab discontinuation

Among the 90 patients with advanced MCC, 52 patients discontinued avelumab treatment. Of the patients who discontinued therapy, 24 initiated treatment with other systemic therapies. Six patients were treated with one systemic therapy, and 18 patients were treated with two systemic therapies (17 carboplatin plus etoposide and 1 cisplatin plus etoposide). The six patients treated with one systemic therapy began a regimen of cisplatin, cyclophosphamide, doxorubicin, pembrolizumab, or topotecan. Of these patients, five had a PR and one was not assessed. Of the 18 individuals who had received two systemic therapies, 5 had a CR, 5 had a PR, and 6 had disease progression. Among patients who received avelumab as 2L+ therapy, only four discontinued avelumab and received a systemic therapy post avelumab treatment. Of the four patients, one received doxorubicin, one received cyclophosphamide, one received topotecan, and one received pembrolizumab. Three of these patients had a PR, and one was not assessed.

## Discussion

This study provides important information about the real-world treatment patterns, outcomes, and clinical characteristics of patients with stage IIIB and IV MCC who initiated avelumab in academic medical centers or their satellite centers in the USA. Despite the severity of disease among the population in this clinical practice study, their positive outcomes were consistent with those observed during clinical trials and in other real-world studies. The median TTD of avelumab therapy was 13.5 months, with a 58% discontinuation rate during the follow-up period. In this study, the rwORR was 73% (39% of patients had a CR), whereas in JAVELIN Merkel 200, the ORR was 33% in previously treated patients with mMCC.^9–11^ Median rwPFS and OS among avelumab-treated patients with advanced MCC were 24.4 and 30.7 months, respectively.

Our study uniquely adds to the body of knowledge of patients with advanced MCC, as a greater proportion of patients included in this study had a more severe baseline ECOG PS than patients in previous clinical trials and real-world studies. This study also included and assessed patients treated with avelumab who had unresectable stage IIIB MCC or stage IV MCC. Comparatively, the JAVELIN Merkel 200 clinical trial only recruited patients with ECOG PS of 0 or 1.^9–11^ The real-world SPEAR-Merkel study examined clinical outcomes in patients with locally-advanced MCC (laMCC) or mMCC, restricted to patients initiating 1L avelumab in a US community oncology setting.^20^ The population in SPEAR-Merkel appears to have had less severe disease than our study’s population, with 14.3% stage IIIB, 21.4% stage IV, 50% ECOG PS 1, 11% ECOG PS 2, and no ECOG PS 3 (compared with this study’s population: 18% stage IIIB, 82% stage IV, 31% ECOG PS 1, 42% ECOG PS 2, and 26% ECOG PS 3). In another real-world study, Levy *et al*^21^ assessed outcomes of patients with advanced MCC, defined as stage IIIB or stage IV, treated with avelumab in four dedicated referral centers in the Netherlands. In their population, 15% of the patients had stage IIIB/laMCC and 85% had stage IV/mMCC, 32% had ECOG PS 0, 59% had ECOG PS 1, and 9% had ECOG PS 2.

While the populations studied in the clinical trial differed from that in our study, both studies still demonstrated the positive outcomes attributable to avelumab treatment. Patients in JAVELIN Merkel 200 demonstrated a clinically meaningful and durable ORR of 33% in previously treated patients with mMCC.^9–11^ Median OS was 12.6 months, and the 3-year OS rate was 32%.^10 12^ In comparison, our overall results in a real-world advanced MCC population were an rwORR of 73.3%, median OS of 30.7 months, and 3-year OS rate of 46%. The rwORR in our study among 2L patients was similar to that of 1L patients (64.7% vs 75.3%), but the rwPFS was much shorter when comparing 2L patients to 1L patients (6.4 months vs 36.1 months), likely driven by the shorter durability of response (4.6 months vs NE) and OS (15.9 months vs 41.7 months) among 2L patients compared with 1L patients. While the significance of these comparisons is limited due to the relatively small sample size of the 2L cohort, the data emphasize the importance of using immunotherapy earlier in the course of patients with advanced MCC.

With the differences in study design and population across the real-world studies, that is, severity of patient population, setting of care, and line of treatment, the outcomes of the real-world studies are not directly comparable. Patients with mMCC in SPEAR-Merkel experienced an rwORR of 63.2% in the USA, while they achieved a response rate of 59% (n=27) among patients with mMCC in Netherlands and an rwORR of 67.6% in patients with stage IV MCC in our study.^20 21^ The median OS of the mMCC population in the SPEAR-Merkel study was 20.2 months, compared with a median OS of 25.8 months for the overall sample (not reported by mMCC) in the Levy *et al* study and 15.9 months for patients with stage IV disease in our study.^20 21^ Focusing on patients with laMCC, rwORR was 66.7% in SPEAR-Merkel compared with a ORR of 50% in patients with laMCC in Levy *et al* and a 100% rwORR in patients with stage IIIB disease in our study.^20 21^ For laMCC, the median OS was not reached in the SPEAR-Merkel study and our study, and it was not reported in the Levy *et al* study. Together these results provide strong evidence that the positive response rates and OS seen in clinical trials translate well into clinical practice for both the stage IV and stage IIIB MCC populations.

Although clinical trials are the gold standard to evaluate the internal validity of the safety and efficacy of drug treatment, they have important limitations. To ensure that heterogeneous patient characteristics do not adulterate the treatment effect being tested, many typical patients with MCC may be excluded from a clinical trial population. For example, patients with an ECOG PS ≥2 were excluded from the JAVELIN Merkel 200 study, and most patients had an ECOG PS of 0. In addition, in JAVELIN Merkel 200, patients were required to have adequate hematologic, hepatic, and renal function. Patients were ineligible for the clinical trial if they had received previous immune checkpoint inhibitor therapy, were receiving concurrent anticancer treatment or systemic treatment with corticosteroids, or had immunosuppression or other clinically significant comorbidities. In contrast, our study examined all patients treated with avelumab, including those with other 1L and 2L+ systemic treatments. These comparisons highlight the important differences in study population between the current real-world study and the pivotal clinical trial of avelumab. While measuring a true treatment effect may subject results to some degree of bias, the real-world design applied in this study enables an evaluation of how avelumab is being used along with its associated effectiveness in a real-world population. Since this population more accurately reflects the patients that oncologists treat in their practices, it provides highly relevant information that supplements the registrational clinical studies, such as the JAVELIN Merkel 200 trial.

There are several limitations to this study. First, the data were obtained by medical chart review, using charts that were recorded for clinical practice rather than for research. Although the study inquiries were developed based on the information usually collected in medical charts, expected information may be missing or excluded during the initial collection or the retrospective transfer of data into collection forms. In addition, there was not a homogenous standard or criteria imposed on the treating provider for assessing tumor response with treatment in the real-world unlike the independently confirmed RECIST-based response in clinical trials. As a result, there may be some variability in the assignment of tumor response not seen in real-world practice. Additionally, all patients observed in this study were treated by physicians practicing at academic medical centers. Academic medical centers may have different operational procedures than non-academic institutions. Patient populations that seek treatment at academic medical centers may also differ from those seeking treatment in other community settings, which may have contributed to our study population being more severely ill than in other studies. Thus, results may not be generalizable to all patients with advanced MCC in the USA or globally. Finally, given the small sample size, this study was unable to generate results for many subgroups of interest. While some inferences can be drawn from very small samples, they cannot be confirmed via statistical analyses. Additionally, there is the possibility of respondent bias, as only a limited number of physicians responded with interest in the study despite a larger outreach to academic medical centers. However, future research could explore these associations further.

The results of this real-world study of patients with stage IIIB and IV MCC in academic centers in the USA showed that avelumab treatment is associated with high response rates that are durable and accompany significantly improved survival, even in patients with relatively poor ECOG PS. The results herein provide an important snapshot of the clinical characteristics, treatment patterns, and real-world clinical outcomes associated with avelumab treatment in a clinical practice setting that augments current evidence from clinical trials and other real-world observational studies.

## Data Availability

Data are available upon reasonable request. Any requests for data by qualified scientific and medical researchers for legitimate research purposes will be subject to Merck’s (CrossRef Funder ID: 10.13039/100009945) Data Sharing Policy. All requests should be submitted in writing to Merck’s data sharing portal (https://www.merckgroup.com/en/research/our-approach-to-research-and-development/healthcare/clinical-trials/commitment-responsible-data-sharing.html). When Merck has a co-research, co-development, or co-marketing or co-promotion agreement, or when the product has been out-licensed, the responsibility for disclosure might be dependent on the agreement between parties. Under these circumstances, Merck will endeavor to gain agreement to share data in response to requests
